# Surgical management of recurrent intrahepatic cholangiocarcinoma: predictors, adjuvant chemotherapy, and surgical therapy for recurrence: A multi‐institutional study by the Kyushu Study Group of Liver Surgery

**DOI:** 10.1002/ags3.12018

**Published:** 2017-07-20

**Authors:** Yo‐ichi Yamashita, Ken Shirabe, Toru Beppu, Susumu Eguchi, Atsushi Nanashima, Masayuki Ohta, Shinichi Ueno, Kazuhiro Kondo, Kenji Kitahara, Masayuki Shiraishi, Yuko Takami, Tomoaki Noritomi, Kohji Okamoto, Yoshito Ogura, Hideo Baba, Hikaru Fujioka

**Affiliations:** ^1^ Kyushu Study Group of Liver Surgery Nagasaki Japan; ^2^ Department of Gastroenterological Surgery Graduate School of Medical Sciences Kumamoto University Kumamoto Japan

**Keywords:** adjuvant chemotherapy, intrahepatic cholangiocarcinoma (ICC), predictor, prognosis, recurrence, repeat resection

## Abstract

Objectives of the present study were to identify predictors of the recurrence of intrahepatic cholangiocarcinoma (ICC), and to evaluate the survival benefit of adjuvant chemotherapy and surgical treatment for ICC recurrence. A multi‐institutional retrospective study was carried out in 356 patients with ICC who underwent curative surgery at one of 14 institutions belonging to the Kyushu Study Group of Liver Surgery. A total of 214 patients (60%) had recurrence. Predictors of ICC recurrence were as follows: positive for pathological intrahepatic metastasis (im), positive for lymph node metastasis (n), positive for pathological lymphatic infiltration (ly), pathological bile duct invasion (b), and tumor size ≥4.4 cm. Adjuvant chemotherapy was given to 120 patients (34%) and, in the patients with im or tumor size ≥4.4 cm, adjuvant chemotherapy showed a survival benefit. Only 37 patients (17%) underwent surgical treatment for ICC recurrence. The surgical treatment resulted in a good 5‐year survival rate (44%), which is similar to the rate obtained by the first operation for primary ICC. Prognosis of patients with primary im after the second operation was significantly worse (5‐year survival 18%) compared to patients without primary im. Primary im+ should be considered a contraindication for surgical treatment for ICC recurrence.

## 1 INTRODUCTION

Intrahepatic cholangiocarcinoma (ICC) is a rare disease; however, it has a relatively highly prevalence in Asia and in the USA, at over 1/100 000 population ratio.^1^ In the USA, the incidence of ICC has increased by 165% over the past 30 years, and surgical resection remains the only curative treatment option.^2^ Surgical results for ICC remain unsatisfactory because of the high rate of recurrence, reported to be 53–71%.^3^, ^4^, ^5^ Several studies showed that cure after resection of ICC is an elusive goal, and there is an increasing awareness of the need for predictors of recurrence such as tumor size ≥5.0 cm,^3^, ^6^ lymph node metastasis,^3^, ^7^ macrovascular invasion,^3^ satellite liver nodules,^7^ and pathological perineural invasion (pn).^7^ To improve patient survival after ICC resection, optimal treatment strategies must be identified for both ICC recurrence and prevention of recurrence such as adjuvant chemotherapy.

The major recurrent focus of ICC has been reported to be the liver, and the rate of ICC recurrence in the liver is approximately 60%.^3^, ^5^ Considerable interest has been paid to various treatment options against ICC recurrence such as surgical treatment,^6^, ^7^, ^8^ chemotherapy,^9^ radiation therapy,^7^ radiofrequency ablation (RFA),^10^, ^11^ and transarterial chemotherapy 12, 13 with various degrees of success. In recent reports, aggressive surgical treatment for ICC recurrence led to good patient survival after recurrence, with a 3‐year overall survival (OS) rate of 25%,^14^ 40%,^6^ and 100%.^7^ However, the efficacy of this strategy remains unclear because of the small numbers of patients who underwent surgical treatment for ICC recurrence in those studies (ie from four to 10 patients).^6^, ^7^, ^14^

In the present study, we attempted to identify predictors of ICC recurrence after curative surgeries, and we evaluated the survival benefit of adjuvant chemotherapy and surgical treatment for ICC recurrence in a multi‐institutional retrospective study conducted by Kyushu Study Group of Liver Surgery for an examination of a large patient sample size.

## 2 METHODS

### 2.1 Patients

Between January 1986 and March 2013, a total of 356 hepatectomies for mass‐forming dominant ICC, confirmed by pathological diagnosis, were carried out at 14 institutions that are members of the Kyushu Study Group of Liver Surgery. Intraductal growth type of ICC, without invasion to the liver parenchyma, was excluded from this study. The medical records of patients in this series were followed until March 2014, with a median follow‐up period of 26 months.

### 2.2 Surgical techniques and follow‐up methods

Details of our surgical techniques and patient follow‐up methods have been reported previously.^15^, ^16^, ^17^ Major hepatectomies with bile duct resection were carried out when bile duct invasion of ICC was suspected to affect the first branch of the hepatic duct. Partial hepatectomies were carried out in cases of peripheral ICC without bile duct invasion. When surgeons believed it would be better to confirm the surgical margins, the resected stump was used for frozen pathology.^15^ The right and left lobes of the liver have different routes of lymphatic drainage, and thus the style of lymph node dissection was different according to tumor location on the right or left lobe.^16^ We generally did not carry out regional lymph node dissection in patients with peripheral ICC without macroscopic swelling lymph nodes.

The application of adjuvant chemotherapy was determined by each physician in charge based on the patient's age, activities of daily life, and the reported presence or absence of poor prognostic factors such as lymph node metastasis (n), pathological lymphatic infiltration (ly), pathological intrahepatic metastasis (im), and pathologically poor ICC differentiation.^15^ Starting in 2006, gemcitabine‐based chemotherapy was generally applied and, from 2008, S‐1 has been the alternative option.^18^, ^19^ Before 2006, oral uracil‐tegafur (UFT) and venous 5‐fluorouracil (5‐FU) were the major options in ICC adjuvant settings.

Indications for surgical treatment for ICC recurrence were determined by each physician or each institutional cancer board. Essentially, surgical treatment was applied only when the patients was considered able to achieve macroscopically curative status by surgical treatment for ICC recurrence, irrespective of the recurrent focuses or the number of recurrences.

### 2.3 Statistical analysis

Continuous variables are expressed as means ± standard deviation (SD) and were compared using Student's *t*‐test. Categorical variables were compared using the χ^2^‐test. Multivariate logistic regression models were used to determine independent predictors of ICC recurrence. Any death that occurred in the hospital after surgery was recorded as a mortality. Complications were evaluated with the Clavien's classification,^20^ and complications with a score of Grade II or more were defined as positive. OS and disease‐free survival (DFS) curves were generated by the Kaplan‐Meier method and compared by the log‐rank test. All analyses were carried out with JMP^®^ Pro 9.0.2 software (SAS Institute, Cary, NC, USA). *P* values <.05 were considered significant.

## 3 RESULTS

### 3.1 Details of ICC recurrence, and its predictors

Intrahepatic cholangiocarcinoma recurrence occurred in 214 patients (60%). First recurrence occurred in 284 focuses such as the liver (104 patients, 37%), lymph nodes (81 patients, 29%), lung (40 patients, 14%), peritoneum (33 patients, 12%), and other organs (26 patients, 9%).

Results of our comparisons of clinicopathological factors associated with ICC recurrence are summarized in Table 1. There were no patient background characteristics that were significantly related to ICC recurrence. As for surgical factors, major hepatectomy (10% vs 82%; *P*=.0009) and R1 operation (20% vs 33%; *P*=.0113) significantly related to ICC recurrence. Many tumor‐related factors significantly related to ICC recurrence; tumor size (4.2±2.7 vs 5.2±3.2 cm, *P*=.0039), multiple tumors (10% vs 22%, *P*=.0030), poorly differentiated (22% vs 36%, *P*=.0130), n (8% vs 32%, *P*<.0001), ly (7% vs 22%, *P*=.0003), im (9% vs 32%, *P*<.0001), pathological portal venous/hepatic venous infiltration (vp/vv) (33% vs 57%, *P*<.0001), pn (16% vs 28%, *P*=.0442), and pathological bile duct invasion (b) (39% vs 59%, *P*=.0014).

**Table 1:** Comparisons of clinicopathological factors associated with ICC recurrence

Variable	Recurrence (–) (n=142)	Recurrence (+) (n=214)	*P* value
Patient background			
Age (years)	67.7±9.6	65.6±10.4	.1928
Male/Female (n)	73/69	125/89	.0562
BMI (kg/m^2^)	22.7±3.3	22.7±3.8	.8872
DM (+) (%)	23 (16%)	25 (12%)	.4493
HBs‐Ag (+) (%)	14 (10%)	19 (9%)	.4443
HCV‐Ab (+) (%)	14 (10%)	28 (13%)	.6074
Alb (g/dL)	4.6±0.6	4.0±0.4	.1851
ICGR‐15 (%)	11.2±5.8	11.6±6.7	.5499
Child A (%)	138 (97%)	198 (93%)	.2535
Surgical factors			
Operation time (min)	451±395	470±175	.5476
Blood loss (g)	1518±135	1405±111	.5195
Transfusion (+) (%)	61 (43%)	97 (45%)	.3134
Major Hx (+) (%)	14 (10%)	175 (82%)	.0009
Lymph node dissection (+) (%)	91 (64%)	143 (67%)	.5940
Bile duct resection (+) (%)	43 (30%)	75 (35%)	.3497
Surgical margin (mm)	10.6±11.5	9.9±11.0	.5331
R1 (%)	29 (20%)	70 (33%)	.0113
Tumor‐related factors			
Tumor size (cm)	4.2±2.7	5.2±3.2	.0039
Solitary/Multiple	128/14	167/47	.0030
Periductal infiltrating type (%)	33 (23%)	66 (31%)	.2009
Poorly diff. (%)	31 (22%)	76 (36%)	.0130
n (+) (%)	11 (8%)	69 (32%)	<.0001
ly (+) (%)	10 (7%)	48 (22%)	.0003
im (+) (%)	13 (9%)	69 (32%)	<.0001
vp/vv (+) (%)	47 (33%)	121 (57%)	<.0001
Pn (+) (%)	23 (16%)	59 (28%)	.0442
b (+) (%)	56 (39%)	126 (59%)	.0014
CEA (ng/mL)	7.8±33.0	252.8±3194.4	.3616
CA19‐9 (IU/L)	5606.3±58 355.1	3379.6±16 423.3	.6113

Alb, Albumin; b, pathological bile duct invasion; BMI, body mass index; CA19‐9, carbohydrate antigen 19‐9; CEA, carcinoembryonic antigen; DM, diabetes mellitus; HBS‐Ag, hepatitis B virus surface antigen; HCV‐Ab, hepatitis C antibody; Hx, hepatectomy; ICC, intrahepatic cholangiocarcinoma; ICGR‐15, indocyanine green retention rate at 15 min; im, pathological intrahepatic metastasis; ly, pathological lymphatic infiltration; n, historical lymph node metastasis; pn, pathological perineural invasion; Poorly diff., poorly differentiated; vp/vv, pathological portal venous/venous infiltration.

Using all significant variables in the univariate analysis, we used multivariate logistic regression models to determine independent predictors of ICC recurrence (Table 2). Receiver operating characteristics (ROC) curve for tumor size identified the cut‐off value for ICC recurrence as 4.4 cm (AUC 0.61585, sensitivity 0.5640, and 1‐specificity 0.2330). Our analysis identified five independent predictors as follows: im (+) (odds ratio [OR] 4.32, *P*=.0006), n (+) (OR 3.56, *P*=.0008), ly (+) (OR 2.84, *P*=.0374), b (+) (OR 1.96, *P*=.0104), and tumor size ≥4.4 cm (OR 1.81, *P*=.0228).

**Table 2:** Independent predictors of ICC recurrence

Variable	Odds ratio	*P* value	95% CI
im (+)	4.32	.0006	1.84‐11.1
n (+)	3.56	.0008	1.67‐8.20
ly (+)	2.84	.0374	1.06‐8.26
b (+)	1.96	.0104	1.17‐3.31
Tumor size ≥ 4.4 cm	1.81	.0228	1.09‐3.04
Poorly dif.	1.56	.1374	0.87‐2.83
vp/vv (+)	1.44	.1892	0.83‐2.50
R1	0.96	.9017	0.51‐1.79
Major Hx (+)	0.88	.6017	0.44‐1.92
Multiple tumors	0.82	.6712	0.32‐2.06
pn (+)	0.75	.4796	0.32‐1.68

b, pathological bile duct invasion; CI, confidence interval; Hx, hepatectomy; ICC, intrahepatic cholangiocarcinoma; Im, pathological intrahepatic metastasis; Ly, pathological lymphatic infiltration; N, pathological lymph node metastasis; Pn, pathological perineural invasion; Poorly dif., poorly differentiated; vp/vv, pathological portal venous/hepatic venous infiltration.

### 3.2 Survival benefit of adjuvant chemotherapy

**Figure 1:**
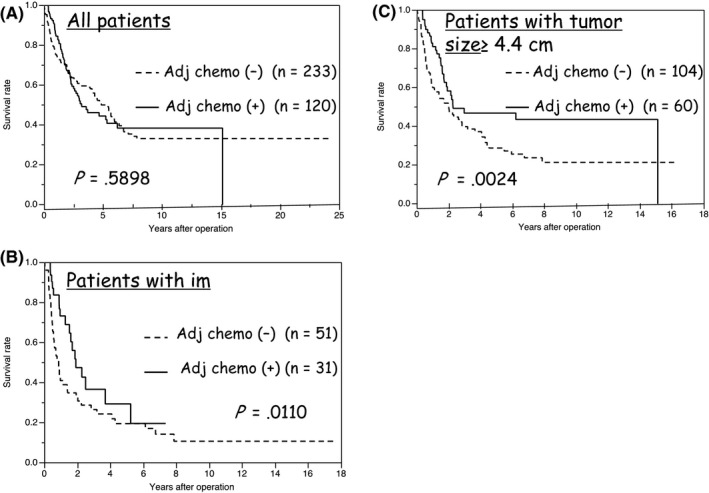
Overall survival (OS) curves of patients after curative operation for intrahepatic cholangiocarcinoma (ICC) according to the presence or absence of adjuvant chemotherapy in (A) all patients, (B) patients with pathological intrahepatic metastasis (im), and (C) patients with tumor size ≥4.4 cm. Adjuvant chemotherapy has survival benefit in patients with im (*P*=.0110) and tumor size ≥4.4 cm (*P*=.0024)

Adjuvant chemotherapy was given to 120 patients (34%), including gemcitabine‐based (67 patients; 56%), S‐1 (19 patients; 16%), and other chemotherapies (34 patients; 28%). Figure 1A demonstrates that adjuvant chemotherapy did not provide a survival benefit in the series of all patients (*P*=.5898). To analyze the survival benefit of adjuvant chemotherapy especially in patients with independent predictors of ICC recurrence, we carried out subgroup analyses concerning patient survival after surgery, and the results are summarized in Table 3. In patients with im (*P*=.0110) and in patients with tumor size ≥4.4 cm (*P*=.0224), adjuvant chemotherapy had a significant survival benefit.

**Table 3:** Survival impact of adjuvant chemotherapy in patients with or without independent predictors of ICC recurrence

Subgroup	1‐year survival (%)	3‐year survival (%)	5‐year survival (%)	*P* value
im (+)				
adj chemo (–) (n=51)	41	26	19	.0110
adj chemo (+) (n=31)	73	37	29	
n (+)				
adj chemo (–) (n=39)	44	23	15	.9763
adj chemo (+) (n=40)	67	5	0	
ly (+)				
adj chemo (–) (n=35)	52	39	29	.6861
adj chemo (+) (n=22)	81	30	20	
b (+)				
adj chemo (–) (n=108)	75	59	45	.6726
adj chemo (+) (n=74)	87	48	42	
≥ 4.4 cm				
adj chemo (–) (n=104)	60	40	29	.0224
adj chemo (+) (n=60)	81	47	47	

adj chemo, adjuvant chemotherapy; b, pathological bile duct invasion; ICC, intrahepatic cholangiocarcinoma; im, pathological intrahepatic metastasis; ly, pathological lymphatic infiltration; n, pathological lymph node metastasis.

### 3.3 Clinical results of surgical treatment for ICC recurrence

Surgical treatment for ICC recurrence was carried out in only 37 patients (17%) with 43 focuses. Various focuses were targeted by the surgical treatment: liver (25 patients, 58%), lymph node (eight patients, 19%), lung (seven patients, 16%), bone (one patient, 2%), adrenal gland (one patient, 2%), and brain (one patient, 2%). There were six patients with two resected focuses: four with liver and lung, and two patients with liver and lymph node focuses. Among 25 patients who underwent hepatectomy, two patients also had ablation therapy such as RFA or microwave coagulation therapy (MCT). Seventeen patients (46%) underwent adjuvant chemotherapy prior to a second surgery. However, this rate was not significantly different from that of the patients who underwent non‐surgical treatment against ICC recurrences (58%, *P*=.1738). Median period between the first and second surgeries was 1.85 (0.56‐9.09) years. Non‐surgical treatment (n=123) for ICC recurrence in our series consisted of chemotherapy (n=93), chemoradiotherapy (n=10), radiotherapy (n=9), ablation therapy (n=6), and transcatheter arterial chemoembolization (n=5).

**Figure 2:**
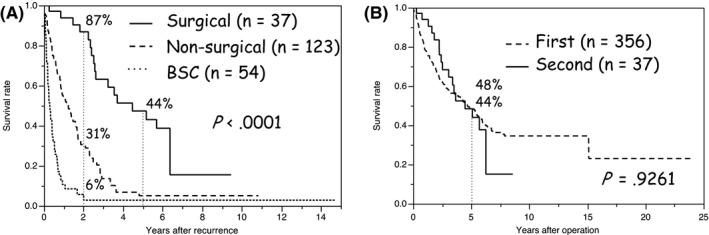
Overall survival (OS) curves of patients with surgical treatment for intrahepatic cholangiocarcinoma (ICC) recurrence after the second operation. (A) Prognosis of patients with surgical treatment is significantly better compared to that of patients with non‐surgical treatment and best supportive care (BSC) (*P*<.0001). (B) There is no significant difference between the survival rate of patients after the first and second operations (*P*=.9261)

Figure 2A demonstrates that the patients who underwent surgical treatment had significantly better survival compared to those with non‐surgical treatment (n=123) and best supportive care (BSC) (n=54, *P*<.0001). The 2‐year survival rate of the patients who underwent surgical treatment was 87% (31% in the non‐surgical treatment group, and 6% in the BSC group), and the 5‐year survival was 44%.

Results of our comparisons of short‐term surgical outcomes between patients with a first operation against primary ICC and those with a second operation against ICC recurrence are summarized in Table 4. The second operations were small‐scale, and operation time was significantly shorter (462±285 vs 304±147 min, *P*=.0020) and blood loss was significantly less (1450±1605 vs 582±603 g, *P*=.0025). Therefore, the measures of short‐term surgical outcome such as mortality (4% vs 0%, *P*<.0001), morbidity (36% vs 5%, *P*<.0001), and duration of hospital stay (31±28 vs 17±9 days, *P*=.0059) were better in the patients with the second operations.

**Table 4:** Comparisons of short‐term surgical outcomes between the first and the second operations for recurrence of ICC

Variable	First operation (n=356)	Second operation (n=37)	*P* value
Operation time (min)	462±285	304±147	.0020
Blood loss (g)	1450±1605	582±603	.0025
Transfusion (+) (%)	158 (44%)	2 (5%)	<.0001
Mortality (%)	13 (4%)	0 (0%)	<.0001
Morbidity (%)	129 (36%)	2 (5%)	<.0001
Hospital stay (days)	31±28	17±9	.0059

ICC, intrahepatic cholangiocarcinoma.

As for patient survival, Figure 2B demonstrates that there was no significant difference between the patients with a first operation (5‐year survival 48%) and those with a second operation (5‐year survival 44%) (*P*=.9261).

**Figure 3:**
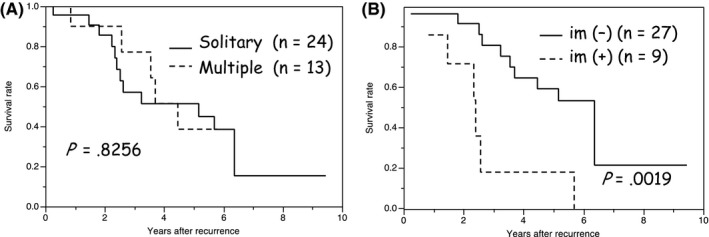
Overall survival (OS) curves of patients with surgical treatment for intrahepatic cholangiocarcinoma (ICC) recurrence after the second operation according to (A) solitary or multiple recurrences and (B) the presence or absence of primary pathological intrahepatic metastasis (im). Prognosis of patients with primary im is significantly worse compared to that without im after the second operation (*P*=.0019)

Prognostic factors for overall survival in the second operations were analyzed, and the results are summarized in Table 5. Only one patient with primary n (1.5%), and two patients with primary ly (4.2%) had a second operation. Factors associated with recurrent ICC such as multiple recurrences, extrahepatic metastasis, and disease‐free interval<1 year cannot predict patient survival after the second operation; however, the factor “im (+)” associated with primary ICCs is the only poor prognostic factor in the second operation. The 3‐year survival rate of patients without primary im (–) was 81%, whereas that of patients with primary im (+) was 18%. The survival curves after the second operation related to solitary or multiple recurrence (*P*=.8256) and to the presence or absence of primary im are provided in Figure 3A and B, respectively.

**Table 5:** Prognostic factors for overall survival in the second operations

Variable	1‐year survival (%)	3‐year survival (%)	5‐year survival (%)	*P* value
Solitary recurrence (n=24)	96	57	51	.8256
Multiple recurrence (n=13)	90	77	39	
Intrahepatic recurrence (n=24)	95	58	37	.3230
Extrahepatic recurrence (n=13)	92	77	77	
DFI ≥1 year (n=8)	92	65	51	.8367
DFI< 1 year (n=29)	100	50	25	
Primary im (–) (n=27)	96	81	59	.0019
Primary im (+) (n=9)	86	18	18	
Primary b (–) (n=15)	100	64	28	.1861
Primary b (+) (n=21)	87	67	67	
Primary tumor size <4.4 cm (n=14)	100	60	36	.1886
Primary tumor size ≥4.4 cm (n=22)	89	70	57	

b, pathological bile duct invasion; DFI, disease‐free interval; im, pathological intrahepatic metastasis.

## 4 DISCUSSION

There have been several reports, including our own, concerning the poor prognostic factors or predictors of recurrence after curative operation for ICC.^3^, ^6^, ^7^, ^15^ In this report, we identified five independent predictors of ICC recurrence such as im (+), n (+), ly (+), b (+), and tumor size ≥4.4 cm. The independent poor prognostic factors in DFS were the same as these five factors (data not shown). Most predictors of ICC recurrence have been tumor‐related factors such as tumor size ≥5.0 cm,^3^, ^6^ n,^3^, ^7^, ^15^, ^17^ macrovascular invasion,^3^ satellite liver nodules,^7^ ly,^15^, ^17^ pn,^7^ and CA19‐9 ≥135 U/mL.^21^ In the present series, median DFS was 6.0 months in the patients with n (n=80) and 8.5 months in the patients with tumor size ≥4.4 cm. Therefore, neoadjuvant chemotherapy would be a potent treatment option for these patients. The use of neoadjuvant chemotherapy can contribute to patient selection for surgery, based on whether or not distant metastasis appears within several months of the chemotherapy. However, as we reported, the preoperative diagnosis for n is difficult even by positron emission tomography–computed tomography, the sensitivity of which is low at 31.2%.^22^

Adjuvant chemotherapy is one of the potent options for the prevention of ICC recurrence. Miura et al. reported the results of adjuvant chemotherapy in 2751 ICC patients, and they analyzed the survival benefit of adjuvant chemotherapy using propensity score‐matched modeling.^9^ In their series, the propensity score‐matched cohort consisted of 1970 patients (985 patients with surgery alone, and 985 patients with surgery + adjuvant chemotherapy), and adjuvant chemotherapy did not provide a survival benefit in all patients (median OS: 20 vs 23 months, *P*=.09). However, in their subgroup analyses of patients with n, T3/T4 tumors, or R1/R2 surgical margins, adjuvant chemotherapy had a significantly better survival benefit. In our present series too, there were significant differences in the patients' characteristics such as n (+) (*P*=.0008) and b (+) (*P*=.0043) between the patients with adjuvant chemotherapy and those without, which indicates that we carried out adjuvant chemotherapy more frequently in the patients who had risk factors for recurrence. Also, in our series, adjuvant chemotherapy provided a survival benefit in the patients with predictors of ICC recurrence such as im (*P*=.0110) and tumor size ≥4.4 cm (*P*=.0224). Despite the encouraging results of adjuvant chemotherapy in patients with predictors of ICC recurrence, Miura et al. did not mention the details of the adjuvant chemotherapy regimen and, also, our findings are based on various chemotherapy regimens. A randomized, multidisciplinary, multinational phase III trial concerning adjuvant chemotherapy with gemcitabine and cisplatin in patients with cholangiocarcinoma (the ACTICCA‐1 trial) is ongoing,^23^ and the results of the ACTICCA‐1 trial will be important information regarding the clinical efficacy of adjuvant chemotherapy in patients with ICC.

In the present series, ICC recurrence occurred in 214 patients (60%), and the major recurrent focuses were the liver (37%), lymph nodes (29%), lung (14%), and peritoneum (12%). Recent reports also mentioned that the major ICC recurrent focus was the liver, and the rate of intrahepatic recurrence was relatively high (60%) compared to our results.^3^, ^5^ In our 81 patients with lymph node recurrence, regional lymph node recurrence occurred in 13 patients only (16%). Therefore, we propose that aggressive and routine lymph node dissection in patients with ICC cannot always contribute to prevention of lymph node recurrence after operation.^15^, ^16^

Although our series is the larger patient population compared to previous reports,^6^, ^7^, ^14^ surgical treatments for ICC recurrence were carried out in 37 patients only (17%). This rate is extremely low compared to that of repeat resection for recurrent hepatocellular carcinoma at 53%.^24^ In previous reports, surgical treatment for ICC recurrence was carried out only in cases of intrahepatic recurrence; however, our series included 18 patients (49%) who underwent surgical treatment for extrahepatic recurrence. Patient prognosis after surgical treatment for extrahepatic ICC recurrence was relatively good (3‐year survival 77%) compared to that for intrahepatic ICC recurrence (3‐year survival 58%), and thus extrahepatic ICC recurrence should not be considered a contraindication for surgical treatment.

We observed that the prognoses of our patients who underwent surgical treatment for ICC recurrence were significantly better compared to patients with non‐surgical treatment or BSC, and 5‐year survival after the second operation reached 44%. This value was almost the same as that of the first operation for primary ICC (48%), and there was no significant difference between the survival curve of patients with first and second operations (Fig 2B). Short‐term surgical results such as mortality (4% vs 0%; *P*<.0001) and morbidity (36% vs 5%; *P*<.0001) in patients with a second operation were significantly better than those in patients with the first operation (Table 4). One of the major reasons for these better short‐term surgical outcomes in patients with a second operation was the early detection of tumor as a result of strict postoperative follow up, and this led to less‐invasive surgeries. Mean tumor size in the second operation was significantly smaller than that in the first operation (2.7 vs 4.8 cm; *P*=.0005). Our results demonstrated that surgical treatment for ICC recurrence is feasible, and provides a survival benefit in patients with ICC recurrence.

The most important and difficult problem is to determine whether ICC recurrence requires surgical treatment. In our series, all patients with a single ICC recurrence (n=24) underwent surgical treatment irrespective of the recurrent focuses. The prognosis of patients with im at the first operation was significantly worse compared to those without im after the second operation (*P*=.0019), and the 3‐year survival was low (18%). Because of those aggressive recurrence patterns, only one patient with primary n (1.5%), and two patients with primary ly (4.2%) had a second operation. However, all three of these patients had a single lymph node recurrence, and they had relatively good survival after the second operation; 6.4 years (alive), 4.5 years (died), and 1.8 years (died).

Limitations of the present study are the study's multi‐institutional retrospective design and the long‐term interval; in addition, our results might be biased as a result of the varying therapeutic policies of the many physicians. However, the number of patients with recurrent ICC who undergo a second operation is quite small and, in this multi‐institutional study, we therefore made it a priority to collect as large a number of such patients as we could. As a result, 37 patients with a second operation were included. This number is by far largest reported of such patients. Of course, prospective studies with the same therapeutic policies for ICC recurrence are necessary to confirm our results.

In conclusion, the rate of ICC recurrence after curative operation in our series was high at 60%. Predictors of ICC recurrence were as follows: im (+), n (+), ly (+), b (+), and tumor size ≥4.4 cm. Adjuvant chemotherapy for patients with im or tumor size ≥4.4 cm would have a survival benefit. Only a few of our patients (17%) underwent surgical treatment for ICC recurrence; however, this treatment led to good 5‐year survival (44%). Primary im (+) should be considered a contraindication for the surgical treatment of ICC recurrence.

## DISCLOSURE

Conflict of Interest: Authors declare no conflicts of interest for this article.
